# 肺上皮-肌上皮癌1例并文献复习

**DOI:** 10.3779/j.issn.1009-3419.2020.02.08

**Published:** 2020-02-20

**Authors:** 亮 陈, 庆姝 李, 广 付, 明建 葛

**Affiliations:** 1 400016 重庆，重庆医科大学附属第一医院胸外科 Department of Thoracic Surgery, The First Affiliated Hospital of Chongqing Medical University, Chongqing 400016, China; 2 400016 重庆，重庆医科大学病理科 Department of Pathology, Chongqing Medical University, Chongqing 400016, China

**Keywords:** 肺上皮-肌上皮癌, 免疫组化, 诊断, 治疗, Lung epithelial-myoepithelial carcinoma, Immunohistochemistry, Diagnosis, Treatment

## Abstract

**背景与目的:**

肺上皮-肌上皮癌（pulmonary epithelial-myoepithelial carcinoma, P-EMC）是一种十分罕见的涎腺型肺肿瘤，尚无标准治疗方案，本文拟分析肺上皮-肌上皮癌的临床特点，探讨肺上皮-肌上皮癌的诊疗方案。

**方法:**

分析1例肺上皮-肌上皮癌患者的临床资料并回顾其他相关临床文献。

**结果:**

上皮细胞免疫组化表达细胞角蛋白，肌上皮细胞免疫组化表达SMA及S-100，二代基因测序以*HRAS*基因突变为主，PD-L1蛋白为阴性。

**结论:**

肺上皮-肌上皮癌大多预后良好，诊断以镜检及免疫组化为主，治疗以手术切除为主，放化疗效果尚不明确。

## Abstract (en)

**Background and objective:**

Pulmonary epithelial-myoepithelial carcinoma is a very rare type of salivary gland lung tumor. No standard treatment plan yet. This article intends to analyze the clinical characteristics of pulmonary epithelial-myoepithelial carcinoma and discuss the diagnosis and treatment of pulmonary epithelial-myoepithelial carcinoma.

**Methods:**

The clinical data of a patient with pulmonary epithelial-myoepithelial carcinoma were analyzed and other relevant clinical literatures were reviewed.

**Results:**

Epithelial cells immunohistochemically expressed cytokeratin and myoepithelial cells immunohistochemically expressed SMA and S-100. The next-generation sequencing was mainly *HRAS* gene mutation and the express of PD-L1 protein was negative.

**Conclusion:**

Most of the patients with Pulmonary epithelial-myoepithelial carcinoma have a good prognosis. Diagnosis mainly depends on microscopic examination and immunohistochemistry. The treatment of pulmonary epithelial-myoepithelial carcinoma is mainly surgical resection. The effect of radiotherapy and chemotherapy is not clear.

肺上皮-肌上皮癌（pulmonary epithelial-myoepithelial carcinoma, P-EMC）是一种十分罕见的涎腺型肺肿瘤，占所有原发性肺癌的0.1%-1%^[1]^。首次于1972年由Donath和Seifert发现并定义为一种临床病理实体肿瘤，其形态学与口腔唾液腺肿瘤相似，具有高度特异性^[2]^。P-EMC主要由嗜酸性胞质的上皮细胞和肌上皮细胞共同组成双层导管样结构，肿瘤多生长于支气管内壁，也可表现为周围性肺癌，属于低度恶性肿瘤^[3]^。现Pubmed关于P-EMC的个案报道约50例，国内报告不足10例，且无前瞻性临床实验指导治疗。本文将报道1例肺上皮-肌上皮癌患者的临床资料，且回顾与本病诊疗相关的其他临床文献。

## 1 材料与方法

### 1.1 临床资料

患者女性，45岁，因“咳嗽1周”于外院行计算机断层扫描（computed tomography, CT）检查发现左下肺异常占位。无基础疾病，既往患有乙型病毒性肝炎4年，未予以抗病毒药物治疗。血清肿瘤标记物（癌胚抗原、鳞状细胞癌抗原、细胞角蛋白19片段）均处于正常范围内。于2019年5月在我院行F18 -氟脱氧葡萄糖正电子发射断层扫描（positron emission computed tomography, PET）提示左肺下叶纵隔旁软组织密度肿块影，约5.4 cm×4.3 cm，肺门区局灶性放射性摄取增高，SUVmax值为13.7，双侧肺门及纵隔未见肿大淋巴结，纤维支气管镜提示左肺下叶基底段支气管见新生物（图 1），取活检提示涎腺组织来源可能性大。遂于2019年5月根据检查结果行左肺下叶切除术，术中发现肿瘤位于左下肺支气管开口处，肿瘤侵犯部分左上肺支气管开口，行左肺下叶支气管袖式切除术，取出左下肺，剖开肿物，大小约2.3 cm×2.0 cm，呈黄白色，质硬，肿瘤远端见一脓腔，后行系统性淋巴结清扫。术后未出现并发症，并于术后第5天出院。术后病理诊断提示肺上皮-肌上皮癌，病理分期为T_3_N_0_M_0_ IIb期[参考国际肺癌研究协会（The International Association for the Study of Lung Cancer, IASLC）肺癌分期第8版]。患者术后未接受任何辅助治疗，随访6个月，未发现局部复发或远处转移。

**1:**
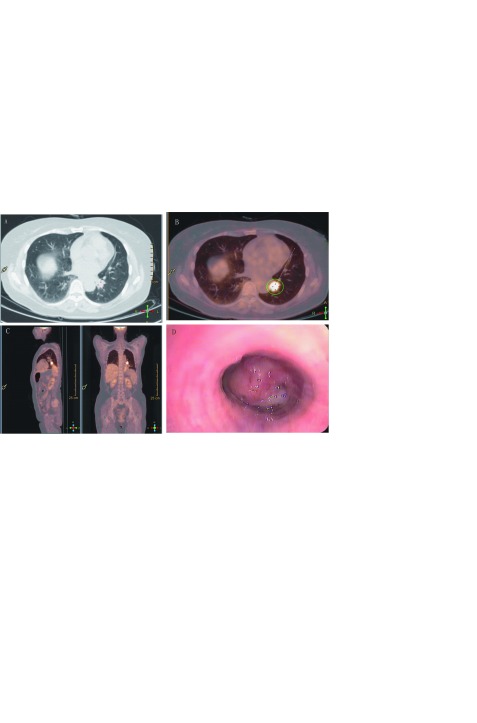
左下肺病变影像学表现及纤维支气管镜检查图像，A：CT平扫提示左肺下叶见一软组织密度肿块影，边界欠清，肿物最大层面大小约5.4 cm×4.3 cm; B：PET/CT提示肺门区局灶性放射性摄取增高，SUVmax值为13.7，SUVmin值为0.4，平均值3.2，肿块大小约2.1 cm×1.8 cm，双侧肺门及纵隔未见肿大淋巴结。C：PET/CT显示头颅、颈部、腹部脏器、四肢等未见放射性摄取异常增高灶; D：纤支镜提示左肺下叶基底段支气管见新生物，外表光滑，见少量脓性分泌物，支气管远端阻塞 Imaging findings and fibrobronchoscopy images of the tumor tissue in left lower. A: CT scan showed a soft tissue density mass in the lower lobe of the left lung, the border was unclear, the maximum size of the mass was about 5.4 cm×4.3 cm; B: PET/CT showed an increased focal radioactive tissue uptake in the hilar area, SUVmax was 13.7, SUVmin was 0.4, MEAN was 3.2, mass size was about 2.1 cm×1.8 cm, and no swelling lymph nodes were found in bilateral hilar and mediastinum. C: PET / CT showed no abnormal and increased radioactive tissue in the skull, neck, abdominal organs, limbs, etc; D: fibrobronchoscopy showed that new organisms were found in basal segment bronchus of the left lung, with a smooth appearance and a small amount of purulent secretion, the distal bronchus is blocked

### 1.2 镜检及免疫组化

以HE染色法可见肿瘤由两种细胞组成，一种是嗜酸性胞质的上皮细胞，位于导管内层，呈立方状，核多居中; 另一种是透明胞质的肌上皮细胞，位于导管外层，多呈类圆形，核偏。两者共同组成导管样结构。免疫组化如下，上皮细胞：TTF-1（-）、CK（+）、AB（+）; 肌上皮细胞：PAS（-）、S-100（+）、SMA（+）、TTF-1（-）、Ki-67 10%（+）（图 2A-H）。介于以上病理学特点，故诊断为肺上皮-肌上皮癌。

**2:**
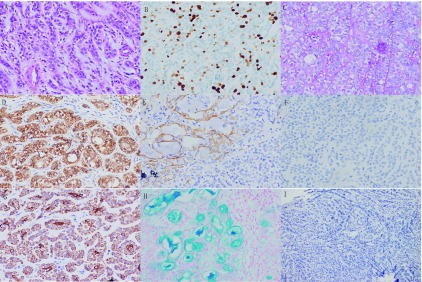
肿瘤组织免疫组化染色，A：（HE染色法，×400）可见上皮细胞和肌上皮细胞的构成腺管双层结构，核异型性不明显，胞质透亮; B：（Ki-67, ×400）散在热点，约10%;C：（PAS, ×400）肌上皮细胞PAS染色呈阴性; D：（S-100, ×400）肌上皮细胞S-100染色呈阳性; E：（SMA, ×400）肌上皮细胞SMA染色呈局灶阳性; F：（TTF-1, ×400）肌上皮细胞及上皮细胞TTF-1染色均为阴性; G：（CK, ×400）上皮细胞CK染色呈阳性; H：（AB, ×400）上皮细胞AB染色呈阳性。I：（PD-L1, ×100）PD-L1蛋白表达为阴性 immunohistochemical of tumor tissue. A: the epithelial cells and myoepithelial cells constitute a bilayer structure of the glandular duct, the nuclear heteromorphism is not obvious, and the cytoplasm is translucent (Haematoxylin and eosin staining, ×400); B: scattered in hot spots, about 10% (Ki-67, ×400); C: myoepithelial cells were negative for PAS staining (PAS, ×400); D: myoepithelial cells were positive for S-100 staining (S-100, ×400); E: myoepithelial cells SMA staining was focally positive (SMA, ×400); F: myoepithelial cells and epithelial cells were negative for TTF-1 staining (TTF-1, ×400); G: epithelial cells were positive for CK staining (CK, ×400); H: epithelial cells AB staining was positive (AB, ×400). I: PD-L1 protein expression was negative (PD-L1, ×400)

### 1.3 二代基因测序及PD-L1表达

以NovaSeq二代测序平台分析肺癌多基因突变，参考GRCh37/hg19基因组，结果为：*HARS*基因上第61号密码子由谷氨酰胺变为精氨酸，*BCOR*基因上第833号密码子由谷氨酰胺变为终止密码子，*TET2*基因上第137号密码子由半胱氨酸变为精氨酸。常规肺癌九基因（包括*EGFR/ALK/ROSI/KRAS/PIK3CA/BRAF/HER2/RET/NRAS*）未检测到基因变异。PD-L1蛋白表达为阴性（图 2-I，VENTANA PD-L1，SP142分析试剂盒），MSI检测结果为稳定型。

## 2 讨论

肺上皮-肌上皮癌是一种罕见的涎腺型肺肿瘤，早期可出现咳嗽、发热、呼吸困难、咯血等症状，个别患者症状类似顽固性哮喘^[4-9]^。既往有多个名称如透明细胞上皮癌、管状实体腺瘤和腺肌上皮瘤等^[4]^。主要诊断依靠病理学形态以及免疫组化，病理学特征包括上皮细胞和肌上皮细胞的腺管双层结构，肌上皮细胞增生和非侵袭性生长，免疫组化为上皮细胞角蛋白阳性，肌上皮细胞S-100和SMA阳性^[7, 9]^。二代基因测序可作为参考。该肿瘤需与腺样囊性癌、肌上皮癌、透明细胞类癌、黏液表皮样癌等相鉴别^[7, 8]^，腺样囊性癌的特征是巢状或条索状上皮细胞被柱状透明间质或粘液性间质包裹，特征性表现为大小不等的筛状结构，这是由于柱状透明间质大量出现在上皮细胞内所致^[10]^，肌上皮癌相较于P-EMC区别在于前者缺乏导管样结构^[6]^，CD56染色阴性可以排除透明细胞类癌^[4]^，粘液表皮样癌内的黏液细胞含有PAS阳性的黏液，故PAS染色阴性可以鉴别。

我们回顾了包括本病例在内的29例临床资料较完整的P-EMC文献^[1, 4-18]^：年龄7岁-76岁，平均53.8岁; 男性8例，女性21例，肿瘤发生率与是否吸烟无明显关系，大小在0.7 cm-12 cm，平均大小2.62 cm，28例患者接受手术治疗，1例患者接受内镜治疗，3例患者出现复发或淋巴结转移，余23例患者随访时间从1个月-78个月不等，均无复发或转移证据。P-EMC的大体标本大多表现为白色质硬结节或肿块，周围可出现囊肿或脓肿，在光镜下都表现为上皮和肌上皮的双层导管样结构，同时周围存在鳞状细胞、透明细胞、梭形细胞等。对于内层的上皮细胞，28例患者细胞角蛋白（包括CK、CK7、CK8/18、AE1/AE3、CK903及CAM5.2）表达为阳性，2例患者S-100为阳性和弱阳性，少数P53、P63、CEA、EMA、AB、CD10及SP-A表达阳性。3例患者S-100为阴性，SMA、VIM未见表达。对于外层的肌上皮细胞，26例患者SMA表达为阳性，20例患者S-100表达为阳性，10例患者细胞角蛋白表达为阳性，3例患者TTF-1表达阳性，少数VIM、P40、P63、 PAS、CD10、calponin、HMWK表达阳性，嗜铬粒蛋白A、NSE、HMB-45、甲状腺球蛋白抗原、Napsin A及desmin未见表达。本例进行了二代基因测序，发现*HARS*基因第61号密码子突变，该结果与Hsieh的研究相似^[19]^，*HARS*基因突变多见于甲状腺癌、唾液腺癌等，通过编码细胞膜GTP酶影响细胞的增殖和抗凋亡。目前尚未有文献报道指出*BCOR*基因和*TET2*基因与肺相关恶性肿瘤有关，少数文献指出前者基因突变与血液系统恶性肿瘤和子宫内膜间质肉瘤有关^[20, 21]^，而后者与淋巴瘤有关^[22]^。故*BCOR*基因和*TET2*基因在P-EMC的诊断、预后、治疗方面的临床意义还有待更多研究证明。在PD-L1检测中，未检测到肿瘤细胞或免疫细胞阳性反应，故免疫抑制剂在P-EMC治疗中价值有待探讨。

目前P-EMC的最佳治疗方式尚未确定，由于肺上皮-肌上皮癌多发于支气管粘膜下层腺体，大多数P-EMC位于支气管，少数P-EMC可生长于外周肺组织，一经诊断，通常选择手术切除。由于P-EMC在大量文献中被描述为低度恶性肿瘤^[4, 6, 9]^，复发和转移的间隔时间较长，是否需淋巴结清扫尚无统一标准。目前有文献提出涎腺型肺肿瘤可发展为高度恶性肿瘤^[19]^，且有个案报道提示肺上皮-肌上皮癌出现淋巴结转移和复发^[7, 14]^。Song报道了1例P-EMC复发病例，文中提到肌上皮所占肿瘤比例高、P27缺失、肿瘤体积大等因素可能会导致复发。考虑到以上不良因素，我们认为P-EMC治疗方式应为肺叶、全肺或袖式支气管切除+系统性淋巴结清扫，对于肺功能不能耐受肺叶切除的患者可采取亚肺叶切除^[8]^。大部分患者手术切除后未予以后续放化疗，Rosenfeld和Cho认为术后不需要化疗，但需严密随访^[2, 7]^，仅有极少数个案报道患者接受术后化疗，由于涎腺型肿瘤大多数发生在腮腺，目前美国国家综合癌症网络（National Comprehensive Cancer Network, NCCN）（2018第1版）针对涎腺型肿瘤提出化疗用于姑息性治疗患者、局部分期较晚的患者，可单独或联用多种药物，如顺铂、环磷酰胺、阿奇霉素、米阔蒽醌、卡铂、长春瑞滨，因远期副作用不推荐中子放疗。Yamazaki报道了1例病例，患者腮腺的涎腺型肿瘤转移至肺部，使用DCF（多西他赛60 mg/m^2^、顺铂60 mg/m^2^、氟尿嘧啶600 mg/m^2^）化疗5个周期后，CT提示肺转移灶消失^[23]^。故目前关于分期较早的P-EMC患者首选治疗方式为手术切除，根据患者是否存在高危因素制定后续治疗方案。

**1:** 各文献患者临床资料 Clinical data of patients in each literature

Author	Age/Sex	Site	Size (cm)	Treatment	Lymph node metastasis	Immunohistochemical detection		Outcome (month)
						Epithelial cell	Myoepithelial cell	
Westacott^[1]^	64/M	RMLL	1.5	Lobectomy	NO	S-100(-); CK(+)	CK5 (+); CK7 (+); S-100 (+); SMA (+); PAS (+)	N/A
Ryska^[4]^	47/M	RUL	N/A	Bronchoscope	N/A	CK(+); SMA(-); VIM(-); S-100(-)	SMA (+); Vim (+); CK (+); S-100 (-); chromogranin A (-); NSE (-); HMB-45 (-)	NED: 13 mo
Konoglou^[6]^	34/M	Bronchus	1.1	Sleeve resection	N/A	CK7(+); CK8/18(+)	SMA (+); S-100 (+); P63 (+)	NED: 24 mo
Song^[7]^	52/F	LLL	12	Lobectomy	NO	CK(+); VIM(-); S-100(-)	SMA (+); S-100 (-)	Recurrence: 117 mo
Song^[7]^	66/M	LUL	1.8	Sleeve resection	NO	CK(+)	SMA (+); S-100 (+)	NED: 75 mo
Song^[7]^	60/M	LUL	0.7	Lobectomy	NO	CK(+)	SMA (+); S-100 (+); TTF-1 (+)	NED: 33 mo
Song^[7]^	61/M	RUL	1.5	Lobectomy	NO	CK(+)	SMA (+); S-100 (+)	NED: 1 mo
Song^[7]^	63/F	Bronchus	0.7	Lobectomy	NO	CK(+)	SMA (+); S-100 (+)	NED: 10 mo
Hagmeyer^[8]^	58/F	RUL	1.5	Wedge resection	NO	CK7(+)	S-100 (+); SMA (+); P63 (+); TTF-1 (+); thyroglobulin (-); Napsin A (-);	NED: 24 mo
Cho^[9]^	51/F	LUL	3	Lobectomy	NO	EMA(+)	S-100 (+); SMA (+)	NED: 16 mo
Shen^[10]^	58/F	LLL	1.3	Lobectomy	NO	CK(+); CEA(+); S-100(+); P63(+); CD10(+)	TTF-1 (+); P40 (+); P63 (+); CK5/6 (+)	NED: 8 mo
Tajima^[11]^	72/F	LLL	4.2	Lobectomy	NO	AE1/AE3(+); p53(-)	AE1/AE3 (-); SMA (+); P63 (+); p53 (-)	NED: 4 mo
Nakashima^[12]^	54/F	RML	1.8	Lobectomy	NO	CK(+)	CK (-); S-100 (+); SMA (+); p63 (+); CK5/6 (+)	NED: 36 mo
Muñoz^[13]^	76/F	RUL	2.7	Lobectomy	NO	CK(+); EMA(+); CEA(+)	SMA (+); S-100 (+); CD10 (+)	N/A
Cha^[14]^	53/F	RML	2.2	Lobectomy	Yes	CK(+)	CK (±); SMA(±); S-100 (+)	N/A
Rosenfeld^[15]^	7/M	LLL	4	Lobectomy	NO	SMA(-); S-100(+)(-)	SMA (+); S-100 (-); CK (+)	NED: 15 mo
Arif^[16]^	57/M	RLL	1.2	Bi-Lobectomy	N/A	AE1/AE3(+); CK7(+); CK903（+）	Calponin (+); S-100 (+)	NED: 9 mo
Nguyen^[17]^	38/M	LLL	5	Lobectomy	NO	CK(+)	CK (+); SMA (+); S-100 (+); TTF-1 (-); GFAP (±); CD117 (±); HMB45 (-)	NED: 4 mo
Nguyen^[17]^	48/M	RUL	2.5	Lobectomy	NO	N/A	N/A	NED: 12 mo
Nguyen^[17]^	52/F	LLL	3	Lobectomy	NO	CK(+)	CK (+); SMA (+)	N/A
Nguyen^[17]^	54/M	RUL	3	Lobectomy	NO	CK(+)	CK (+); SMA (+)	NED: 12 mo
Nguyen^[17]^	56/F	LMB	4.2	Pneumo	Yes	CK(+)	CK (+); SMA (+); S-100 (+); TTF-1 (-); GFAP (±); CD117 (±); HMB45 (-)	NED: 4 mo
Chang^[18]^	54/F	RLL	2.6	Wedge resection	NO	CK7(+); CAM 5.2(+); EMA(+); SP-A(+); TTF-1(+); CK20(-); HMWK(-)	HMWK (+); SMA (+); S-100 (+); calponin (+); p63 (+); desmin (-)	NED: 31 mo
Chang^[18]^	62/F	LLL	2.6	Wedge resection	NO			NED: 14 mo
Chang^[18]^	58/F	RML	2.6	Wedge resection	NO			NED: 13 mo
Chang^[18]^	57/F	LUL	0.8	Wedge resection	NO			NED: 78 mo
Chang^[18]^	52/F	RUL	1.2	Wedge resection	NO			NED: 5 mo
This study	45/F	LLL	2.3	Sleeve resection	NO	TTF-1(-)，CK(+)，AB(+);	PAS (-); S-100(+); SMA (+); TTF-1(-)	NED: 6 mo
F: female; M: male; RUL: right upper lobe; RML: right middle lobe; RMLL: right middle and lower lung; RLL: right lower lobe; LUL: left upper lobe; LLL: left lower lobe; N/A: not available; mo: month; NED: no evidence of disease; Pneumo: Pneumonectomy; LMB: Left main bronchus.								

综上所述，P-EMC是一种罕见的低度恶性肿瘤，预后良好，诊断以镜下发现上皮细胞和肌上皮细胞的腺管双层结构为依据，治疗方式以手术切除为主，放化疗治疗效果尚不明确，受限于病例数较少，缺乏临床对照研究，需要进一步研究证明放化疗是否影响患者预后。
